# Sonographic Features of Uterine Arteriovenous Malformation: A Case Series

**DOI:** 10.3390/diagnostics14090873

**Published:** 2024-04-23

**Authors:** Dhammapoj Jeerakornpassawat, Charuwan Tantipalakorn, Sirinart Sirilert, Theera Tongsong

**Affiliations:** Department of Obstetrics and Gynecology, Faculty of Medicine, Chiang Mai University, Chiang Mai 50200, Thailand

**Keywords:** arteriovenous malformation (AVM), computed tomography angiography (CTA), ultrasound, uterine artery embolization (UAE), uterus

## Abstract

Uterine arteriovenous malformation (AVM) is very rare but potentially life-threatening. Early and accurate diagnosis is the cornerstone of its management. The objective of this study is to encourage sonographers to become familiar with a variety of grayscale sonographic features, facilitating rapid recognition of the patterns and prompting them to apply color flow Doppler for a diagnosis of uterine AVM and possible further investigations or interventions. We present six cases of uterine AVM presenting with abnormal uterine bleeding at varying degrees of severity, from abnormal menstruation to life-threatening bleeding following curettage. All initially provided some clues of uterine AVM upon grayscale ultrasound, leading to the application of color Doppler flow to support a diagnosis, with confirmation using abdominal computer tomography angiography (CTA) in most cases, resulting in definitive treatment using uterine artery embolization or other interventions. Most importantly, this study provides various sonographic features of uterine AVM, such as appearances of small tubular structures, spongy patterns, a conceptive-product-like appearance, and spaghetti-like patterns. Hopefully, familiarity with these sonographic features can facilitate practitioners to make an early diagnosis, leading to proper further investigation and intervention, and to prevent serious complications from potentially being caused by this subtle but very serious disorder.

## 1. Introduction

Uterine arteriovenous malformation (AVM) is a pathologic phenomenon described as a faulty short circuit of the bloodstream between the arterial and venous supply of the uterus, characterized by the bloodstream having an unusually high velocity, turning the vessels into a vascular fistula [1]. It may be congenital or acquired (traumatic AVM), representing a number of small AV fistulae in the myometrium, and may have unilateral or bilateral uterine artery feeders. It is a rare condition, but including subtle cases of under-reporting, its prevalence may be as high as 4.5% [2]. The presentation of uterine AVMs can vary from asymptomatic to various degrees of vaginal bleeding, which can be life-threatening and necessitate hospitalization and blood transfusions [3,4,5,6,7,8,9]. Additionally, subtle cases of uterine AVM can be unexpectedly provoked or triggered by a simple gynecologic procedure such as intrauterine curettage, which can cause excessive bleeding and may possibly lead to life-threatening situations [10].

Several non-invasive diagnostic modalities have been used to diagnose uterine AVM, such as computed tomography angiography (CTA), magnetic resonance imaging (MRI), color flow Doppler ultrasound, three-dimensional (3D) ultrasound, power Doppler angiography, and amplitude-mode color Doppler ultrasound. Nevertheless, this study exclusively emphasizes its sonographic features as essential primary tools for the diagnosis of uterine AVM, instead of back-up tools, like computed tomography angiography (CTA), or therapeutic intervention, as focused on in several studies [11,12,13,14,15,16,17]. This is due to the fact that the effectiveness of diagnosis leading to definitive treatment is primarily based on the first significant clues obtained from routine ultrasound in actual practice. Accordingly, this article places importance on the sonographic features of two-dimensional grayscale ultrasound, which prompt sonographers to apply color flow Doppler for a diagnosis of uterine AVM and possible further investigations or management. The objective of this study is to help practitioners improve their diagnostic performance based on two-dimensional ultrasound and color flow Doppler ultrasound, with further back-up investigations as necessary. Importantly, this study alerts sonographers not to overlook non-specific abnormal lesions in the uterus, which can result in delayed diagnosis and treatment, and also not to carry out unnecessary investigations. The diagnostic criteria in all the cases presented here were primarily based on sonographic features, both subjective, with a rich vascular network observed in the myometrium with the use of color Doppler imaging, and objective, with a high peak systolic velocity of greater than 20 cm/sec recorded in the vascular web [1].

## 2. Case 1 (Figure 1)

A 32-year-old woman, P1011, presented with abnormal vaginal bleeding 4 h prior to admission, with no other symptoms. Her last pregnancy, 3 years prior to this admission, was a term pregnancy complicated by postpartum hemorrhage due to a retained piece of the placenta, and manual removal of the placenta was performed. Her underlying disease was controlled hyperthyroidism (euthyroid state, normal thyroid function test). The general physical examination was normal. Pelvic examination revealed no structural abnormalities, except active bleeding per the cervical os. The basic laboratory work-up and hematologic study showed a normal hemoglobin level of 12.1 g/dL. Her serum beta-hCG was less than 0.2 mIU/mL. Two-dimensional ultrasound and color Doppler examination indicated uterine AVM. Abdominal CTA indicated uterine AVM.

**Figure 1:**
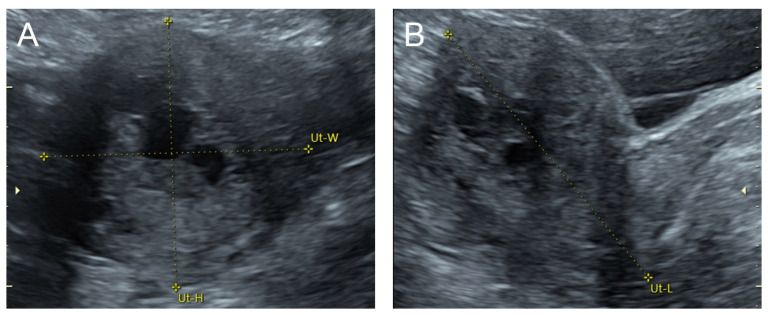

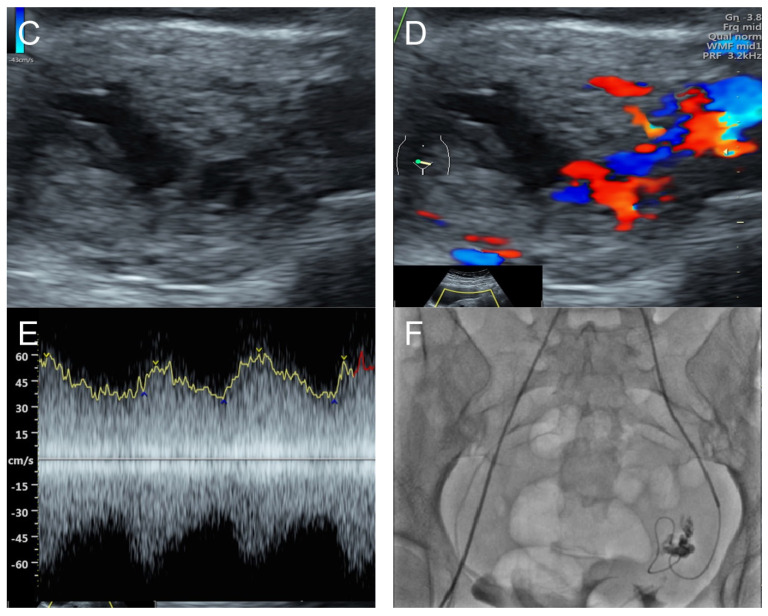
(**A**–**C**) Transabdominal grayscale ultrasound: cross-sectional, sagittal, and oblique scans of the uterus showed heterogeneous soft tissue content, like pieces of conceptive product in the uterine cavity; ill-defined endometrial–myometrial interface; hypoechoic lacunae varying in size in the non-specific tissue content, mainly localized at the left anterior wall. (**D**) Color flow mapping with a relatively high pulse repetition frequency of 3.2 kHz (applied to the same image (**C**)) showed hyper-vascularized lesions in the myometrium; multidirectional flow, mainly localized at the left anterior wall; and some cystic spaces of no flow, indicating lysed blood in the cavity. (**E**) Spectral Doppler ultrasound showed a high peak systolic velocity (approximately 60 cm/s). The sonographic diagnosis was uterine AVM. The main differential diagnoses were incomplete abortion (conceptive products) and gestational trophoblastic disease. (**F**) CTA during uterine embolization revealed hypervascularity and tortuous arterial anatomy enhancing a dilated vascular pouch overlying the endometrium of the uterus with feeding via the bilateral uterine arteries and draining via the internal iliac veins, confirming uterine AVM; low blood content in the uterine cavity without evidence of active contrast extravasation.

*Management:* Initial therapy included blood transfusion, tranexamic acid administration, and intrauterine tamponade with a Foley catheter balloon, resulting in less but continued bleeding. Emergency uterine artery embolization on both sides, using glue (Glubran 2 cyanoacrylate) mixed with Lipiodol (iodized oil), was successfully performed. The follow-up abdominal CTA at 8 weeks after the procedure showed no residual AVM, and the patient was healthy with no longer abnormal bleeding at 3-year follow-up.

## 3. Case 2 (Figure 2)

An 80-year-old woman, P5003, 30 years post-menopause, presented with new-onset active vaginal bleeding 1 day prior to admission, with no other symptoms and no history of abnormal uterine bleeding. Her four full-term pregnancies ended with normal vaginal delivery, and the last one involved cesarean section with tubal sterilization (35 years ago). One spontaneous abortion (~45 years before) required curettage. She had no underlying disease. The general physical examination revealed mild pale conjunctiva. Pelvic examination revealed an atrophic vagina and active bleeding per the cervical os. The basic laboratory work-up and hematologic study were normal, except for a hemoglobin level of 9.0 g/dL. Two-dimensional ultrasound and color Doppler examination indicated uterine AVM. Abdominal CTA indicated uterine AVM.

**Figure 2:**
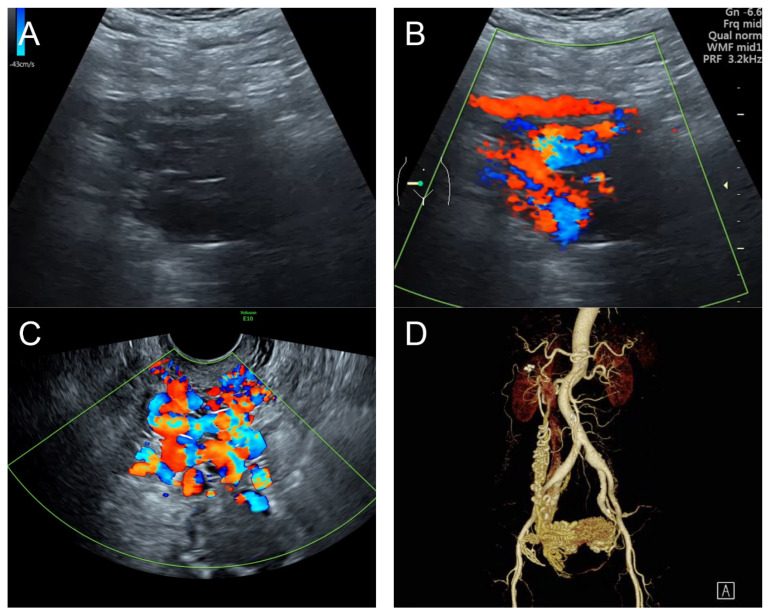
(**A**) Transabdominal grayscale ultrasound: transverse scans of suprapubic area showed suboptimal-quality image of the uterus, which displayed ill-defined soft tissue mass, non-visualized endometrial lining, hypoechoic areas of the uterus. No normal architecture of the uterus could be demonstrated. (**B**) Color flow mapping (the same area of figure (**A**)) showed markedly vascularized uterus, involving throughout the uterus intense vascularity with a chaotic, multidirectional flow. (**C**) Transvaginal color Doppler ultrasound showed markedly vascularized uterus, involving throughout the uterus intense vascularity with a chaotic, multidirectional flow. (**D**) Abdominal CTA revealed innumerable tortuous dilated vessels in the pelvic cavity, along the entire uterine wall. The lesions were fed by multiple arterial feeders, which were displayed as tortuous dilated arteries of the bilateral uterine arteries, right ovarian artery, and right inferior epigastric artery. Multiple draining veins demonstrated early venous opacification, namely the bilateral internal iliac veins and right ovarian vein. The CTA findings confirmed uterine AVM.

*Management:* Initial therapy included fluid resuscitation, blood transfusion, and tranexamic acid administration. Transcatheter uterine embolization of the bilateral uterine arteries, right ovarian artery, and right inferior epigastric artery was successfully performed, without immediate post-operative complications. The follow-up abdominal CTA at 8 weeks after the procedure showed no residual AVM, and the patient was healthy with no further abnormal bleeding at 2.5-year follow-up.

## 4. Case 3 (Figure 3)

A 55-year-old woman, P2012, presented with abnormal vaginal bleeding 3 months prior to admission, with no other symptoms. Her two term pregnancies ended with normal vaginal delivery. One abortion was spontaneous, without dilation and curettage. She entered menopause at the age of 52. She had used depot medroxyprogesterone acetate between 30 and 50 years of age and used no hormonal therapy during post-menopause. The general physical examination revealed mild pale conjunctiva. Pelvic examination revealed active bleeding per the cervical os and the perception of pulsations and a turbulent flow at the left posterior fornix. The basic laboratory work-up and hematologic study were normal, with a hemoglobin level of 11.8 g/dL. Two-dimensional ultrasound and color Doppler examination indicated uterine AVM. Abdominal CTA indicated uterine AVM.

**Figure 3:**
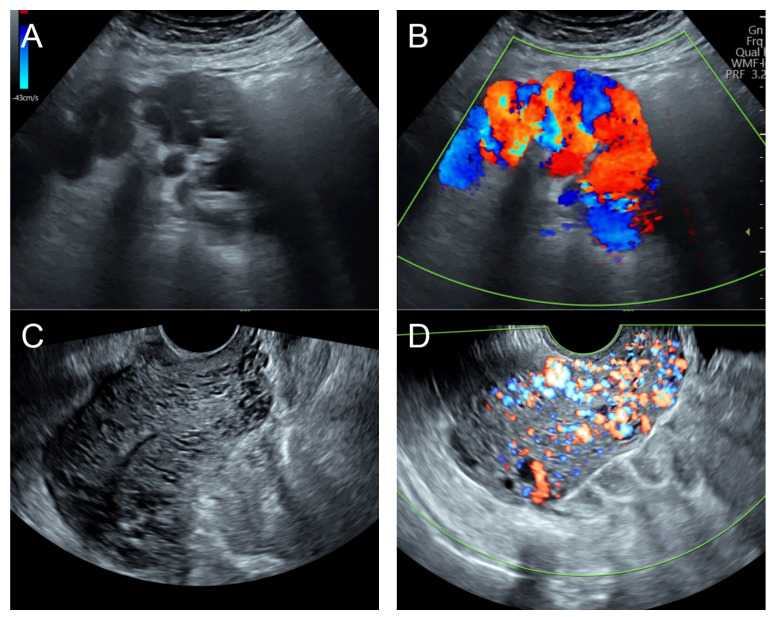

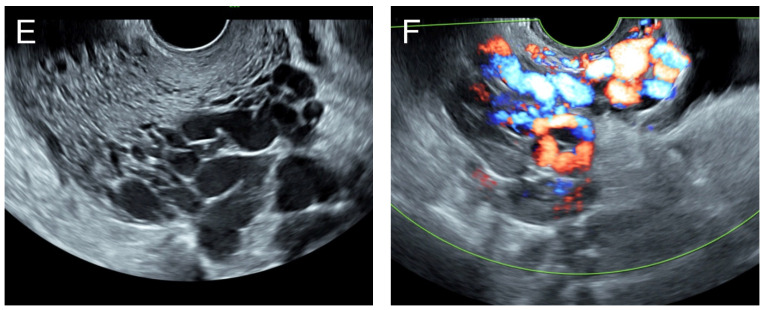
(**A**,**B**) Grayscale ultrasound and color flow mapping transabdominal sagittal scans of the left lower quadrant showed a markedly enlarged and tortuous course of the left uterine artery, with a high peak systolic velocity of 60 cm/s in spectral Doppler waveforms. (**C**,**D**) Sagittal scans of the uterus in transvaginal grayscale ultrasound and color flow mapping showed numerous anechoic/hypoechoic tiny tubular structures within the myometrium, which tended to head to the endometrium in the direction perpendicular to the endometrial line. These tubular structures were scattered all over the uterus and strongly vascularized throughout the uterus. (**E**,**F**) Sagittal scans of the left adnexa in transvaginal grayscale ultrasound and color flow mapping showed a markedly enlarged and tortuous course of the left uterine artery.

*Management:* Initial therapy included blood transfusion and intrauterine tamponade with a Foley catheter balloon, resulting in less but continued bleeding. Abdominal CTA confirmed uterine AVM, and transcatheter embolization of both uterine arteries, using glue mixed with lipiodol, was performed, resulting in a reduction in bleeding. However, the follow-up abdominal CTA at 8 weeks after the procedure still showed significant residual AVM, and the patient still had abnormal uterine bleeding. Finally, a subtotal hysterectomy with bilateral salpingo-oophorectomy with resection of the AVM was performed, with satisfactory outcomes. Pathological examination confirmed the diagnosis of uterine AVM. Note that a subtotal hysterectomy, instead of a total hysterectomy, was carried out in order to minimize the risk of intraoperative excessive bleeding due to abundant vascularization in the lower uterine segment. On follow-up, the patient was healthy with no further abnormal bleeding at 2 years after the hysterectomy.

## 5. Case 4 (Figure 4)

A 20-year-old woman, P0010, presented with an episode of acute heavy menstrual bleeding. Her vital signs on presentation were within the normal limits. This was her second visit to our clinic in one month with similar complaints. On her previous visit, expectant management with NSAIDs and tranexamic acid was provided. Physical examination revealed mild pale conjunctiva, and otherwise, she was within the normal limits. Pelvic examination revealed a normal genital tract, except that there was active uterine bleeding from the cervical os, which was closed. Her basic laboratory results were also within the normal limits, except that she experienced a significant drop in hemoglobin from 10.8 g/dL on the previous visit to 8.9 g/dL. Her urine pregnancy test was negative. Two-dimensional ultrasound and color Doppler examination indicated uterine AVM.

**Figure 4:**
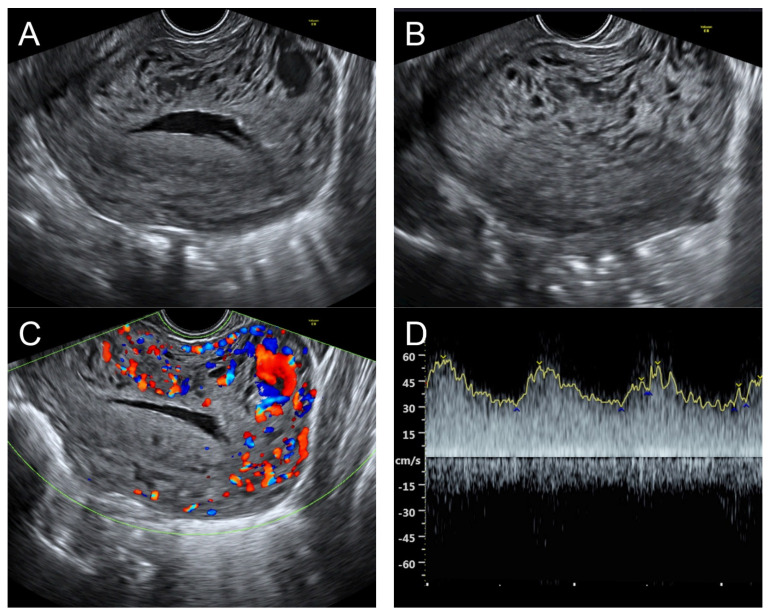
(**A**) Transvaginal ultrasound: sagittal scan of the uterus showed several dense small anechoic/hypoechoic tubular structures, mainly running to the endometrium, scattered throughout the anterior wall of the uterus; a thin endometrium; no other specific lesions of the uterus. There was anechoic fluid collection in the uterine cavity. (**B**) Cross-sectional scan of the fundus showed lesions with poorly defined outlines, containing several dense small anechoic/hypoechoic tubular structures, packed in the anterior wall. (**C**) Color flow mapping showed hyper-vascular areas in the myometrium, without well-defined lesion borders; multidirectional flow mainly localized at the anterior wall and the fundus. The main differential diagnosis was adenomyosis or gestational trophoblastic disease. (**D**) Spectral Doppler showed a high peak systolic velocity (~50 cm/s) with a low resistance index of 0.3. Note that in differentiating from adenomyosis, which sometime shows multiple minute anechoic or spongy-like areas but not lacunar lakes or tortuous vessels, adenomyosis is characterized by the absence of flow or minimal flow or by the presence of straight, scattered vessels traversing a hypertrophic myometrium [18,19], typically not containing a high flow peak systolic velocity, as seen in AMV, as mentioned earlier.

*Management:* Initial therapy included blood transfusion, tranexamic acid administration, oral misoprostol 400 mg every 4 h with 3 doses, and intrauterine tamponade with a Foley catheter balloon, resulting in a significant reduction in bleeding. The tamponade was removed after 24 h of indwelling. Combined low-dose contraceptive pills were used for contraception and the prevention of abnormal uterine bleeding. The patient did not have heavy menstrual bleeding during the three months of follow-up. Note that the diagnosis of this case was exclusively based on the sonographic criteria described earlier, without confirmatory CTA.

## 6. Case 5 (Figure 5)

A 30-year-old woman, P1001, was referred to our hospital because of profuse vaginal bleeding after curettage due to abnormal uterine bleeding one hour before referral. She had a history of one previous low-transverse cesarean section. Her hemoglobin level on admission was 5.6 g/dL, and she had been transfused 4 units of packed red blood cells. Her vital signs were unstable, with a blood pressure of 80/40 mmHg and a pulse rate 120 bpm. Pelvic examination revealed no structural abnormalities, except for active bleeding per the cervical os, which was closed. The other basic laboratory studies had normal results. Her urine pregnancy test was negative. Two-dimensional ultrasound and color Doppler examination indicated uterine AVM. Uterine artery embolization was not available.

**Figure 5:**
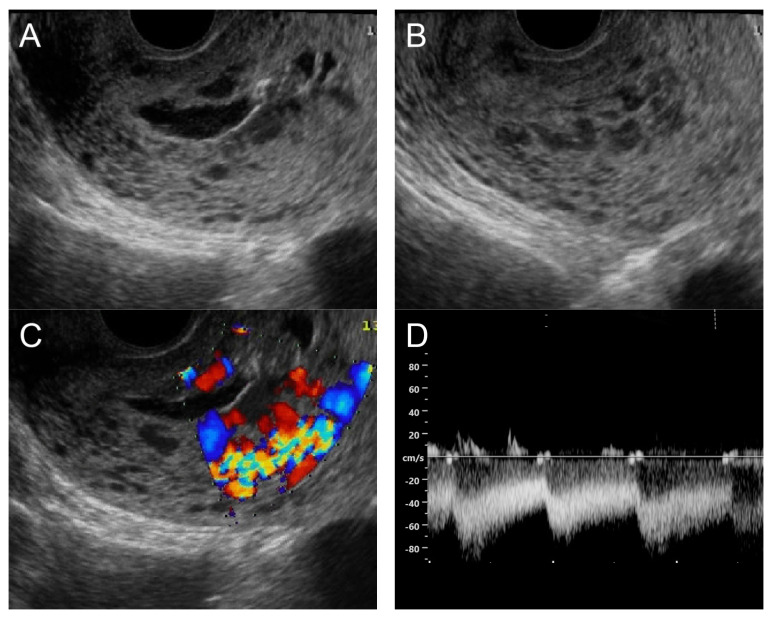
(**A**,**B**) Transvaginal ultrasound: sagittal scans of the uterus showed several small anechoic/hypoechoic cysts, giving a spongy pattern and varying in size, throughout the myometrium; no other specific lesions of the uterus. The uterine cavity was lined with a thin endometrium and filled with anechoic fluid, probably lysed blood. (**C**) Color flow mapping showed hyper-vascularization in the cystic spaces, as seen in figure (**A**,**B**), throughout the myometrium and a multidirectional chaotic flow. The main differential diagnosis was adenomyosis or gestational trophoblastic disease. (**D**) Spectral Doppler showed a high peak systolic velocity (~70 cm/s) with a low resistance index of 0.2.

*Management:* Initial therapy included fluid resuscitation, blood transfusion, trans-rectal misoprostol at 800 mg, and intrauterine tamponade with a Foley catheter balloon, resulting in a reduction in bleeding, but significant bleeding still persisted. A transabdominal total hysterectomy was successfully performed, with satisfactory outcomes. The operative findings and pathological examination confirmed the diagnosis of uterine AVM. The patient was healthy and asymptomatic at the 6-week post-operative check-up. However, unfortunately, she was lost to follow-up after this point.

## 7. Case 6 (Figure 6)

A 28-year-old woman, P0010, presented with excessive vaginal bleeding on and off for six months prior to this visit. She had a history of one previous spontaneous complete abortion without dilatation and curettage. She was healthy, without any medical underlying disease. Pelvic examination revealed no structural abnormalities, except for active bleeding per the cervical os, which was closed. The laboratory studies had normal results, with a hemoglobin level of 12.0 g/dL. Her urine pregnancy test was negative. Two-dimensional ultrasound and color Doppler examination indicated uterine AVM. Uterine artery embolization was not available.

**Figure 6:**
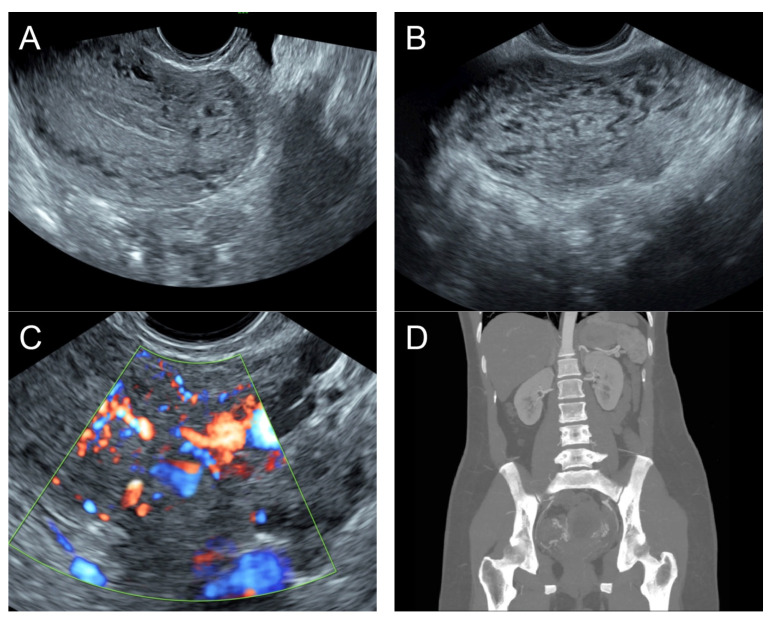
(**A**) Transvaginal ultrasound: sagittal scan of the uterus showed heterogeneous echoes of the myometrium; a dilated arcuate artery at the posterior wall; several small anechoic/hypoechoic spongy structures within the myometrium, mainly at the anterior wall; no other specific lesions of the uterus. The endometrium appears normal. (**B**) Cross-sectional scan of the fundus showed several small anechoic/hypoechoic tubular structures or a spaghetti-like appearance throughout the myometrium. (**C**) Color flow mapping showed hyper-vascular areas in the myometrium, without well-defined lesion borders; multidirectional flow mainly localized at the anterior wall. The main differential diagnosis was adenomyosis or gestational trophoblastic disease. (**D**) Abdominal CTA revealed hypervascularity and tortuous arterial anatomy enhancing dilated vessels of the uterus, with bilateral uterine arteries as the feeding arteries, confirming uterine AVM.

*Management:* On admission, the patient was successfully treated with medications, consisting of oral contraceptive pills (ethinyl estradiol 50 mcg) three times a day, tranexamic acid, and a concomitant Foley balloon tamponade on the day of admission. The tamponade was removed after 48 h of indwelling. Abdominal CTA on day 3 of admission was suggestive of uterine AVM. Continuous low-dose contraceptive pills were prescribed. However, the patient was lost to follow-up after three months of treatment.

## 8. Comment

Uterine AVM is a potentially life-threatening disorder, and its diagnosis requires a high degree of suspicion on the part of practitioners or gynecologists. Prompt diagnosis could avoid hysterectomy and therefore preserve fertility due to the availability of several conservative alternatives. The learning points gained from this report to facilitate early diagnosis and avoid delayed management may be summarized as follows:

- Uterine AVM must be listed in the differential diagnoses in patients presenting with acute and heavy vaginal bleeding, especially in cases with a previous history of uterine instrumentation. Nevertheless, typical images, as presented in this study, should prompt clinicians to consider the possibility of AVM even if there is no previous history of uterine injury. This is because a minority of cases with uterine AVM have no obvious predisposing factors. The pathogenesis in such cases is unclear, but congenital errors in the embryogenesis of the primitive vascular structures may play a role in the development of AVM [20,21].
- The familiarity of the sonographic features of two-dimensional grayscale ultrasound suggestive of uterine AVM is most important since they are the first clues which prompt sonographers to confirm AVM using color flow Doppler ultrasound in the first place, while additional modalities like CTA or three-dimensional ultrasound may be needed in some selected cases.
- Typically, grayscale ultrasound commonly reveals subtle myometrial heterogeneity or small anechoic spaces, varying in size and distributed in the myometrium, or a thickened uterine wall with mixed echogenic ill-defined areas and numerous small tubular structures or spongy anechoic or hypoechoic areas within the myometrium.
- On grayscale ultrasound, the main differential diagnoses include retained pieces of conceptive products, adenomyosis, degenerative leiomyoma, and gestational trophoblastic disease.
- Color Doppler as well as spectral Doppler ultrasound of the pelvis are the most important tools in differentiating uterine AVM from other uterine disorders.
- Abdominal CTA is very helpful for confirmation and should be considered the non-invasive gold standard of diagnosis in some selected cases.
- Uterine artery embolization is an appropriate minimally invasive intervention in most cases of uterine AVM, especially in cases with the need for fertility preservation.
- Patient counseling issue: This study emphasizes the accurate preoperative diagnosis of uterine AVM, which is very important for patient counseling, in which potential serious complications, the choices of treatment, the options for fertility preservation in some cases, and the benefit of CTA in selective cases must be taken into consideration.

*Research Implications:* Various clinical and sonographic features in this study may serve as data sources for case accumulation in the literature and future meta-analyses to develop the management guidelines. Actually, future studies still need to compare the sonographic features of uterine AVM with other uterine pathologies as a control group, like partial moles, adenomyosis, etc.

## Data Availability

The data in this report are available from the corresponding authors upon request.
